# The Perchlorate Reduction Genomic Island: Mechanisms and Pathways of Evolution by Horizontal Gene Transfer

**DOI:** 10.1186/s12864-015-2011-5

**Published:** 2015-10-26

**Authors:** Ryan A. Melnyk, John D. Coates

**Affiliations:** Department of Plant and Microbial Biology and Energy Biosciences Institute, University of California, Berkeley, CA 94720 USA; Physical Biosciences Division, Lawrence Berkeley National Laboratory, Berkeley, CA USA

## Abstract

**Background:**

Perchlorate is a widely distributed anion that is toxic to humans, but serves as a valuable electron acceptor for several lineages of bacteria. The ability to utilize perchlorate is conferred by a horizontally transferred piece of DNA called the perchlorate reduction genomic island (PRI).

**Methods:**

We compared genomes of perchlorate reducers using phylogenomics, SNP mapping, and differences in genomic architecture to interrogate the evolutionary history of perchlorate respiration.

**Results:**

Here we report on the PRI of 13 genomes of perchlorate-reducing bacteria from four different classes of Phylum Proteobacteria (the Alpha-, Beta-, Gamma- and Epsilonproteobacteria). Among the different phylogenetic classes, the island varies considerably in genetic content as well as in its putative mechanism and location of integration. However, the islands of the densely sampled genera *Azospira* and *Magnetospirillum* have striking nucleotide identity despite divergent genomes, implying horizontal transfer and positive selection within narrow phylogenetic taxa. We also assess the phylogenetic origin of accessory genes in the various incarnations of the island, which can be traced to chromosomal paralogs from phylogenetically similar organisms.

**Conclusion:**

These observations suggest a complex phylogenetic history where the island is rarely transferred at the class level but undergoes frequent and continuous transfer within narrow phylogenetic groups. This restricted transfer is seen directly by the independent integration of near-identical islands within a genus and indirectly due to the acquisition of lineage-specific accessory genes. The genomic reversibility of perchlorate reduction may present a unique equilibrium for a metabolism that confers a competitive advantage only in the presence of an electron acceptor, which although widely distributed, is generally present at low concentrations in nature.

**Electronic supplementary material:**

The online version of this article (doi:10.1186/s12864-015-2011-5) contains supplementary material, which is available to authorized users.

## Background

Although widely distributed in the environment, perchlorate (ClO_4_^−^) is generally present at low concentrations (~14 ng.L^−1^ across the continental US, Alaska, and Puerto Rico) [1]. While it is toxic to humans, it serves as a valuable electron acceptor for several lineages of bacteria. The canonical microbial metabolism of perchlorate depends on its reduction to chlorite (ClO_2_^−^) in the bacterial periplasm by perchlorate reductase, and the dismutation of chlorite to chloride and molecular oxygen [2]. The biogenic molecular oxygen is not released extracellulary but rather is reduced by the same microorganism, generally through the use of a high-affinity cytochrome *cbb*_*3*_ oxidase. The microaerobic portion of this respiration means that dissimilatory perchlorate-reducing bacteria (DPRB) uniquely straddle the realms of both aerobic and anaerobic respirations [2]. Genetic studies in the model DPRB *Azospira suillum* PS have revealed that many of the genes essential for this metabolism are located on a genomic island [3] previously identified in a comparative genomic analysis of four DPRB genomes [4]. This genomic island, designated the perchlorate reduction genomic island (PRI), has clear signatures of horizontal gene transfer (HGT).

Since our original identification of the PRI, we have accumulated 9 additional genome sequences of DPRB, including isolates from the Epsilon- and Gammaproteobacteria [5, 6]. Additionally, we have increased the depth of sampling in certain genera, as there are now three sequenced isolates in the *Azospira* genus, and two in the *Magnetospirillum* genus [7]. In this study, we set out to increase our understanding of the mechanisms of the evolution of PRI. Specifically, we sought to identify mechanisms of integration and excision of the PRI, in addition to uncovering the evolutionary history of the individual genes in the PRI. In answering these questions, we made observations about the evolution of the PRI that led us to propose new explanations for the origin, evolution, and ecological significance of bacterial perchlorate respiration.

## Methods

All genome sequences are publicly available on the IMG database. The accession numbers (or locus tags, for IMG sequences) for all genes discussed in the manuscript can be found in Additional file 1: Table S2. For the genomes sequenced in this study, sequencing methodology and statistical information can be found on the IMG database. Briefly, genomes were sequenced using 150 bp paired end reads on an Illumina HiSeq 2000 and the resulting reads were then assembled using velvet.

### MLSA analysis

**Fig. 1:**
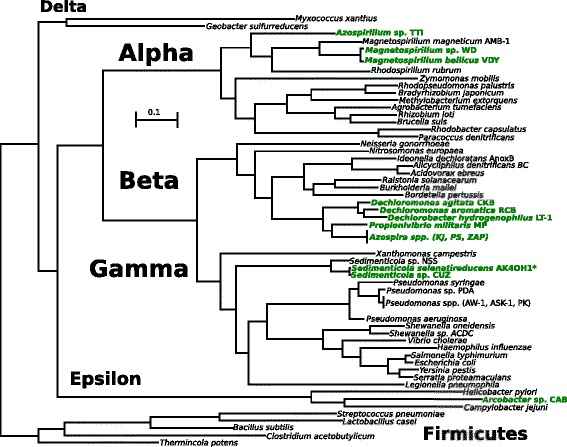
Phylogeny of the Proteobacteria highlighting DPRB. The dataset used to generate this phylogeny consisted of 90 taxa and 92 conserved orthologous genes, resulting in an alignment of 13066 amino acid positions. Using the LG substitution model, this tree was determined to be the most likely of 100 independently calculated trees, and all nodes were fully supported over 100 bootstrap replicates. DPRB are bolded and in green. The scale bar indicates the branch length that correspond to 0.1 amino acid substitutions per site

**Fig. 2:**
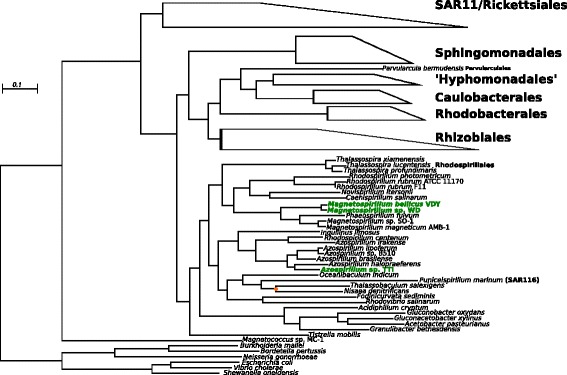
A tree detailing the relationships within the Alphaproteobacteria, focusing on the Rhodospirillales order. The alignment consisted of 9766 amino acids across 80 genes from 96 taxa and the phylogenetic tree was calculated under the LG substitution matrix using 100 bootstraps and 50 inferences to pick the best tree. All nodes had 100 % bootstrap support with the exception of the node marked with the orange dot. DPRB are bolded and in green. The scale bar indicates the branch length that correspond to 0.1 amino acid substitutions per site

**Fig. 3:**
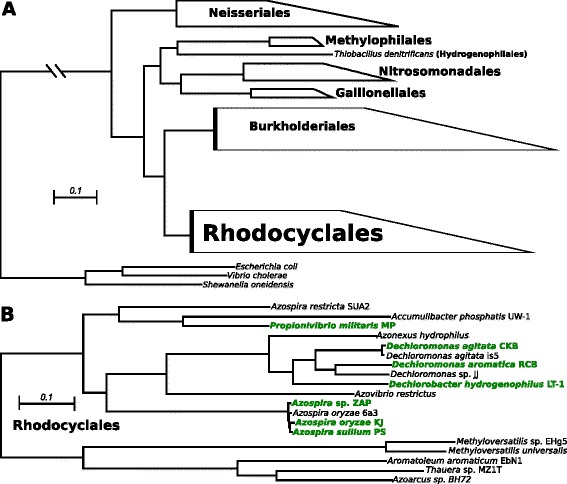
Phylogenetic trees depicting the Betaproteobacteria class and the Rhodocyclales, the only DPRB-containing order. **a** The alignment consisted of 21601 non-redundant amino acid positions from 147 orthologous genes. The tree was calculated using the LG substitution matrix and overlaying the results of 100 bootstrap replicates over the most likely tree of 50 independent inferences. **b** The Rhodocyclales subtree is rooted to taxa from a sister clade within the same order, which were chosen based on their position in the tree made for all Betaproteobacteria (i.e. Fig. 5A). The alignment used to construct this tree consisted of 139587 amino acid positions from 229 genes across 19 taxa. The tree was calculated under the LG + F substitution matrix and was generated by drawing 100 bootstrap replicates on the best tree chosen from 50 independent maximum-likelihood inferences. For both trees, all nodes had 100 % bootstrap support and the scale bar indicates the branch lench corresponding to 0.1 substitutions per site. DPRB are bolded and in green

Well-known taxa were chosen for illustrative purposes in the Proteobacterial dataset (Fig. 1). We eliminated redundancy at the genus level in the Alpha- and Betaproteobacteria datasets prior to enriching the Rhodospirillaceae and Rhodocyclacease families with draft proteomes from the Joint Genome Institute’s IMG database (Figs 2 and 3). These two families are home to all of the perchlorate-reducing isolates in the Alpha- and Betaproteobacteria classes; thus, we hoped that denser taxon sampling of these families would increase our resolution of relationships among perchlorate-reducing bacteria and also provide insight into the origin of accessory genes in the PRI. In each of the datasets, we included well-established outgroups (e.g. using Firmicutes as outgroups for all Proteobacteria).

To detect orthologs, we used an all-vs-all pairwise BLASTP approach to identify orthologs between each possible pair of proteomes in each dataset encode using inParanoid [8]. Each pairwise inParanoid output was used as input to the QuickParanoid clustering algorithm to identify clusters of homologous proteins (http://pl.postech.ac.kr/QuickParanoid/). We designated orthologs as those clusters that had exactly one representative from each organism in each dataset (i.e. Proteobacteria, Alphaproteobacteria, Betaproteobacteria, or Rhodocyclaceae). Each set of orthologs was aligned using MUSCLE [9], and the resulting alignments were processed using Gblocks [10] running parameters designed to maximize retention of all informative sites [11]. The alignments were concatenated and converted to Phylip format and an appropriate model of amino acid substitution was selected using ProtTest 3 [12]. Using the best model and the previously selected outgroup, phylogenetic trees were generated using RAxML [13]. A tree topology was calculated using a maximum-likelihood method for choosing the best tree from a number of independent inferences. A number of bootstrap replicates were performed as well on each dataset, and these were drawn onto the most likely tree to generate support values for each node. This method is described thoroughly in the RAxML manual. The amino acid substitution model and the number of independent inferences and bootstraps are given in the figure legends for each of the four datasets.

In order to simplify the viewing of the Alphaproteobacteria and Betaproteobacteria trees, we collapsed taxonomic groups at the order level based on the published taxonomy. In general, there was robust support for all taxonomic orders with the exception of three species from order Rhodobacterales in the Alphaproteobacteria (*Hirschia baltica, Hyphomonas neptunium, and Maricaulis maris*), which claded with the Caulobacterales. This was previously observed for the genome of the species *Hyphomonas neptunium*, which was assigned to the Rhodobacterales based on 16S phylogeny, but groups with the Caulobacterales on the basis of housekeeping gene sequences [14]. These three species are all members of family Hyphomonadaceae, which we have depicted in our tree as the order-level classification ‘Hyphomonadales’ following a previously set precedent [15]. We note that this is currently not an official taxonomic grouping, but our analysis suggests that these species should reside in a sister group to the Caulobacterales or perhaps within that order itself.

The program Mauve was used to generate whole-genome alignments of the *Azospira* draft genomes (ZAP, KJ, and 6a3) to the finished reference genome of *Azospira suillum* PS, in addition to aligning contigs from the two *Magnetospirillum* draft assemblies (VDY and WD).

### Individual gene phylogenies

For the phylogenies of independent genes, we first used a HMMER3 protein similarity search to identify similar proteins to the query of interest in our entire HAMAP/IMG dataset [16]. We then took all similar proteins and clustered them with a 90 % identity threshold using CD-HIT to reduce the complexity of the dataset [17], prior to generating an alignment as before, using MUSCLE and Gblocks. We then calculated a best maximum-likelihood tree from 10 independent inferences using RAxML [13]. We then viewed the tree using Dendroscope [18], and chose an ingroup containing the sequences from the PRI and an appropriate outgroup. The selected sequences were aligned and curated again as before, using MUSCLE and Gblocks. A best amino acid model was determined using ProtTest 3 and a phylogenetic tree with support values was calculated as before using RAxML and the best tree/bootstrap method. In several instances, RogueNaRok (https://github.com/aberer/RogueNaRok) was used to remove rogue taxa that led to conflicting bootstrap topologies and thus ambiguous phylogenetic signals, prior to tree recalculation.

### Availability of Supporting Data

The data sets supporting the results of this article are included within the article and its additional files. All genome sequences have been uploaded to the IMG database and will be made publicly available upon acceptance of the manuscript. The accession numbers (or locus tags, for IMG sequences) for all genes discussed in the manuscript can be found in Additional file 1: Table S2.

### Ethics

This work did not require humans, human data, or animals, thus a statement of ethics is not required.

## Results

### Perchlorate/chlorate reducers are restricted to certain subclades within the Proteobacteria

All of the genomes used in this study are publically available and their accession numbers along with associated references for their phenotypic descriptions are provided in Additional file 2: Table S1. To assess the phylogenetic relationships between DPRB and other bacteria, we adopted a multi-locus sequence analysis (MLSA) method that relies upon an in-house pipeline for identifying as many orthologs as possible within a given group of bacteria. While MLSA methods do not fully recapitulate the reticulate and piecewise nature of bacterial genome evolution and speciation [19], they are generally satisfactory for resolving a consensus pattern of species divergence [20], particularly when many “taxonomically complete” loci are used [21].

Although DPRB isolated to date are scattered throughout the Proteobacteria (Fig. 1), their presence within classes seems to be restricted. In the Alphaproteobacteria, DPRB are only found in the family Rhodospirillaceae (Fig. 2), while in the Betaproteobacteria, they are present in only one subgroup within the Rhodocyclaceae (Fig. 3). This has been observed since the advent of molecular phylogeny in the context of DPRB [2], but our phylogenetic analysis confirms and strengthens these inferences by making use of the entire genome, rather than the 16S genes of DPRB isolates [22, 23]. DPRB isolates from the Epsilonproteobacteria and Gammaproteobacteria have only recently been characterized and sequenced [5, 6], thus it is impossible to make sense of their distribution within their respective taxonomic classes until more strains have been isolated.

### PRIs have a diverse, yet functionally similar, ‘core’ set of genes

**Fig. 4:**
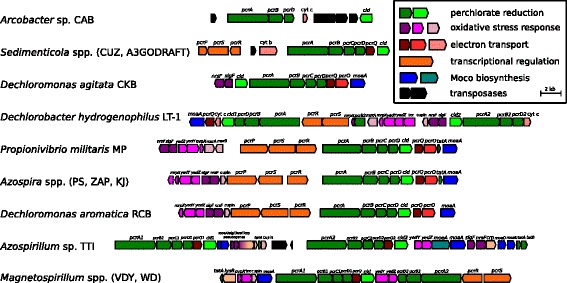
The PRI core from the 13 DPRB genomes. Genes are colored according to functional groups and labeled with gene names, rather than locus tags. Locus tags can be found in Table S2

The original identification of the PRI consisted of 4 separate island ‘cores’, which were defined by beginning at the *pcrA* and *cld* genes and using pairwise BLAST analysis to detect genes shared by more than one DPRB [4]. With the sequencing of additional PRIs, more classes of accessory genes have been observed, although they tend to fit within four main functional groups: transcriptional regulation, electron transport, oxidative stress resistance, and molybdenum cofactor biogenesis (Fig. 4).

Transcriptional regulation in response to environmental flux was identified as a possible role for genes in the PRI, specifically via a histidine kinase signaling pathway [4]. This was confirmed using genetics, as *pcrPSR* are each independently required for perchlorate reduction in *A. suillum* PS [3]. The *pcrSR* genes in *Propionivibrio militaris* MP [22], *Dechloromonas aromatica* RCB [24], and *Azospira* spp. form a monophyletic group with the *pcrSR* genes from *Magnetospirillum* spp. PRIs. The orientation of *pcrS* and *pcrR* on different coding strands is conserved in this group, but *Sedimenticola* spp. and *Dechlorobacter hydrogenophilus* LT-1 each have histidine kinase systems with unique genomic and domain organization (Fig. 4). Nevertheless, we have used the *pcrPSR* nomenclature developed in PS for these organisms to illustrate that they may play a similar role in transcriptional regulation. In the Alphaproteobacteria DPRB, there is the possibility of other transcriptional regulators involved with perchlorate reduction as the PRI of *Azospirillum* sp. TTI contains a *crp*-type regulator and the PRI of *Magnetospirillum* spp. contain a *lysR*-type regulator (Fig. 4).

The exact mechanism of electron transport from the inner membrane to the periplasmic perchlorate reductase is unknown. PcrC is a soluble tetraheme cytochrome previously thought to be an important part of perchlorate reductase function [25]. The *pcrC* gene is essential for perchlorate reduction in *A. suillum* PS [3], although its complete absence in the genome of *Arcobacter* sp. CAB indicates that it is not universally required for perchlorate reductase function [5]. The electron transport mechanism is difficult to infer from genomic observation alone, as PRIs have many genes that could be involved in transporting reducing equivalents (red highlighted genes, Fig. 4). Two genes occur in multiple PRIs: *pcrQ*, which encodes a quinol dehydrogenase tetraheme cytochrome similar to well-characterized proteins such as CymA, NrfH, and NapC [26], and *pcrO,* which is homologous to the gamma subunit of ethylbenzene dehydrogenase [27].

Oxidative stress defense has recently emerged as an important fitness determinant during (per)chlorate reduction [28], likely due to the inadvertent accumulation of the side product hypochlorite from chlorite dismutation [29]. The key genes in this functional group are the *sigF*/*nrsF* sigma factor/anti-sigma factor system, the putative methionine sulfoxide reductase genes *yedYZ*, and the methionine-rich peptide gene *mrpX,* which form a periplasmic methionine-cycling mechanism to detoxify hypochlorite [30]. New PRIs have reiterated the importance of this function, as *P. militaris* MP and *D. hydrogenophilus* LT-1 also contain homologs of the methionine sulfoxide reductase gene *msrA* and *msrB* (Fig. 4). However, unlike the cytoplasmic housekeeping version of these genes, they are predicted to be exported to the periplasm, where the bulk of perchlorate-associated oxidative stress is likely to occur.

Perchlorate reductase is part of the diverse DMSO reductase protein family and thus contains a molybdenum ion bound by two pterin guanine dinucleotide groups, also known as the molybdenum cofactor, or Moco [31, 32]. Within the PRI of every DPRB in the Alpha- and Betaproteobacteria, there is a *moaA* homolog (Fig. 4). MoaA is a radical SAM-dependent enzyme which catalyzes the first step in pterin synthesis from GTP [31, 33]. All of the known DPRB isolates contain a homolog of *moaA* elsewhere in the genome, however, the PRI *moaA* gene appears to be important for perchlorate reduction in minimal media at least in the case of *A. suillum* PS [3].

### PRIs are incorporated at distinct genomic sites, even within a family

**Fig. 5:**
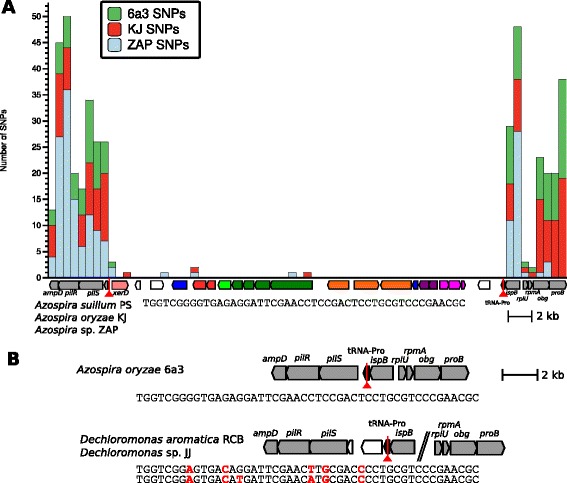
Diagram of the PRI and flanking regions in *Azospira* spp*.* **a** A plot of the SNP abundance of *Azospira* spp. 6a3, KJ, and ZAP relative to the finished PS genome seqence. Because 6a3 lacks the PRI, SNPs were counted over a 500-bp window and overlaid on the PRI and flanking regions from PS, as individual columns in the histogram. The nucleotide sequence of the 48-bp site of insertion in the proline tRNA is located below the gene diagram. **b** This image contains the flanking regions in the non-DPRB *A. oryzae* 6a3 and two *Dechloromonas* spp. demonstrating the synteny of the flanking regions prior to insertion of the PRI. Here, the nucleotide sequence of the putative site of integration within the proline tRNA is shown for all strains (indicated by the red line on the genetic diagrams). 6a3 has an identical insertion site to the DPRB *Azospira* spp., but the *Dechloromonas* spp. have several SNPs

The exact boundaries of the PRI outside of the core were not delineated in its initial observation, but genes associated with mobilization were found outside of the core [4]. Variability in mobilization genes was even observed between PRIs with similar cores, suggesting that the core genes can be found in various types of mobile elements [4]. In the complete genome of the canonical DPRB, *A. suillum* PS, the PRI core genes are located close to a proline tRNA gene [4]. Upon closer inspection, this gene includes a 48-bp sequence that is also found at the other end of the PRI (Fig. 5). Using this region as a scaffold, we were able to assemble a contig with the same genomic organization in the other two *Azospira* DPRB strains, KJ and ZAP [34]. We noticed that the genes outside of these repeat regions were orthologs detected in all Rhodocyclaceae (e.g. *ispB*, *pilSR*) so we looked at their organization in other strains. These flanking genes frequently form a contiguous region in both other DPRB and non-perchlorate reducing strains such as *Azospira oryzae* 6a3, confirming that the PRI was integrated into the proline tRNA (Fig. 5). In other Rhodocyclaceae, however, this operon is not intact but has had a different genomic island incorporated, suggesting that this specific tRNA is a frequent target of mobile elements. Outside of the PRI core but within the boundary formed by the direct repeats is a site-specific recombinase homologous to *xerD* [35, 36], thus providing a possible mechanism for PRI integration.

The boundaries of the entire PRI were unclear in the genome of *D. aromatica* RCB, which lacked homologs of the non-core genes from the *Azospira* spp. PRI. However, using our ortholog detection approach to compare the genome of strain RCB to its close relative, *Dechloromonas* sp. JJ, we were able to delineate the exact boundaries of the PRI in RCB. The RCB PRI is much larger, spanning 145 kB and containing genes for plasmid replication and conjugation. This structure denotes an integrative and conjugative element, or ICE, and may be capable of catalyzing its own excision, transmission and perhaps replication [37].

Due to the fragmentary nature of draft genome assemblies, we were unable to confidently map the location of the PRI insertion in *P. militaris* MP or *D. agitata* CKB. In *D. hydrogenophilus* LT-1, the PRI is inserted in a region with conserved synteny in other Rhodocyclaceae. However, there are no obvious ‘scars’ or evidence for a mechanism of integration and no close non-perchlorate reducing relative to LT-1, so the history of its PRI is unclear. The *D. agitata* CKB PRI is located on its own relatively small contig, but re-mapping of paired-end reads to the contigs using bowtie indicates that it is flanked by near-identical insertion sequences, thus forming a composite transposon. This organization was seen in chlorate-reducing bacteria, where the genetic ‘cargo’ for the metabolism is located between two insertion sequences, which enable its mobilization [38].

### The PRIs from several isolates within the genera Azospira and Magnetospirillum are the result of recent HGT and positive selection

The conservation of the PRI in the three DPRB of the four sequenced *Azospira* species and its integration into an identical location in all three (Fig. 5), might be suggestive of one acquisition event prior to the divergence of the four strains, followed by loss of the PRI in strain 6a3. However, the nucleotide comparisons between the *Azospira* strains do not support such a history. By mapping SNPs in the three *Azospira* draft sequences (strains KJ, ZAP, and 6a3) to the reference strain PS genome, we see that the PRI in KJ and ZAP has very few SNPs, especially when compared to the conserved genes outside the PRI, which have mutation rates roughly average for what is expected from the genome as a whole (Fig. 5, Table 1). The lack of nucleotide divergence between the three PRIs, even at synonymous sites and intergenic regions indicates that the PRI was acquired independently and recently by the three extant *Azospira* DPRB isolates. Of note is that two of the six SNPs in the PRI result in amino acid substitutions in the sequence of PcrA, the initial reductase in the perchlorate reduction pathway (I431L in *Azospira* sp. ZAP, Y736H in *Azospira* sp. KJ), raising the possibility that parts of the PRI may be evolving under positive selection.

**Table 1:** SNP frequency in the PRI and genomes of *Azospira* spp*.*

	PRI SNPs	genome SNPs	PRI frequency	genome frequency
**KJ vs. PS**	3	74295	0.01 %	1.97 %
**ZAP vs. PS**	3	66033	0.01 %	1.75 %
**6a3 vs. PS**	n/a	53194	n/a	1.41 %

### PcrAB and Cld: Monophyletic anchors of an ancestral PRI

**Fig. 6:**
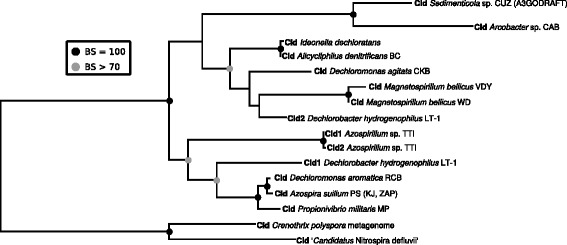
Phylogenetic tree of Cld sequences. Phylogenetic tree constructed using an alignment of Cld sequences from DPRB and rooted to two sequences predicted to be periplasmic Cld sequences not found in DPRB. The alignment used to construct this tree had 255 amino acid positions. The best tree was calculated using the WAG substitution model and was chosen from 200 independent inferences, and bootstrap support values were calculated from 1000 bootstrap replicates

**Fig. 7:**
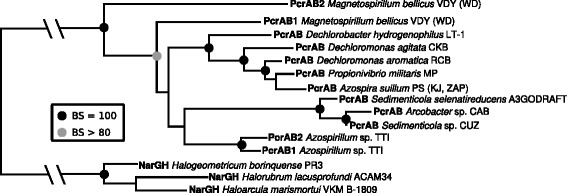
Phylogenetic tree of PcrA sequences*.* Phylogenetic tree constructed using a concatenation of the PcrA and PcrB sequences from several DPRB and rooted to the periplasmic NarGH sequences from several Archaea. PcrA (907 positions) and PcrB (330 positions) alignments were constructed separately, then concatenated to generate the RAxML input. The LG + F matrix was used for the PcrA portion of the alignment and the WAG matrix was used for the PcrB segment. The best tree was chosen from 200 independent inferences and the bootstrap values were calculated from 1000 replicates

Although the *pcrAB* and *cld* genes found in perchlorate-reducing bacteria have phylogenies suggestive of horizontal gene transfer, they both are largely monophyletic and thus support a single ancestral PRI (Figs 6 and 7). The only exception to this is the co-option of the *cld* gene sequence in the formation of chlorate-reduction transposons (e.g. in *Ideonella dechloratans* and *Alicycliphilus denitrificans*); however, these *cld* alleles likely came from the PRI of an existing DPRB [38]. We rooted the Cld sequences from DPRB and two chlorate-reducing bacteria by using a metagenomic sequence and the Cld sequence from ‘*Candidatus* Nitrospira defluvii’ [38, 39]. A more comprehensive phylogeny of Cld sequences indicates that these sequences are intermediates between DPRB Clds and the broadly distributed cytoplasmic Cld [38, 40]. The PcrAB sequences were concatenated and used to construct an alignment rooted to three sequences of Archaeal periplasmic nitrate reductases [41, 42], which are closely related to extant PcrAB sequences [38].

## Discussion

### Why only certain clades?

Although perchlorate reduction is distributed broadly among four different classes within the Proteobacteria, the taxonomic scope of isolates within those classes is narrow. The reason for this is unknown, but is plausibly the result of the interplay of multivariate factors. First, it is very likely that isolation and enrichment techniques are biased for a certain type of DPRB, likely a copiotroph that has a fast growth rate. It may be that these organisms represent only one of several microbial ecological strategies that make use of perchlorate reduction. Indeed, it has previously been observed that altering traditional enrichment parameters such as pH, temperature, or salinity is sufficient to alter the perchlorate reducers isolated [5, 22]. Additionally, 16S or metagenomics-based analyses of perchlorate-reducing communities may uncover a taxonomically richer set of organisms than those currently present in DPRB isolate collections; this avenue has recently been taken by several research groups studying perchlorate-removing bioreactor communities [43, 44].

An organism may also require additional traits in order to become a successful perchlorate-reducer. Generally speaking, perchlorate reducers are motile aquatic organisms, can utilize a variety of electron donors (and acceptors), and are generally non-fermentative facultative anaerobes [2]. It may be that only certain taxonomic groups such as Rhodocyclaceae and Rhodospirillaceae are prepared to be successful in environments such as these, and thus these groups harbor the most cosmopolitan DPRB.

Another possibility is that this taxonomic restriction is enforced by the mechanism of PRI integration. The *Azospira* PRI is integrated at a very specific site in a tRNA, and the exact sequence of the site is not conserved in other Rhodocyclaceae. This specificity may mean that *Azospira* has an advantage in certain environments, where it can acquire a version of the PRI easily through site-specific integration at a neutral genomic location. Furthermore, our phylogenetic analysis indicates that accessory genes from the PRI are often related to genes found in the host organism. We hypothesize that this streamlines PRI integration with host metabolism, specifically in the realms of regulation and electron transport. Specificity of both integration mechanisms and accessory genes results in a positive feedback loop where a PRI becomes more and more optimized for a given lineage. This may in practice reduce DPRB diversity, as an *Azospira* strain that has acquired a PRI tailored for its needs will be much more competitive than an organism attempting to piece together a PRI *de novo*.

### Why is perchlorate reduction so obviously associated with horizontal gene transfer?

The lack of the island in *A. oryzae* sp. 6a3 and the nucleotide identity shared in the PRIs of perchlorate-reducing *Azospira* spp. indicates that perchlorate reduction is not a conserved function of this genus, nor is it for any taxonomic group we have studied. Additionally, the ubiquitous presence of HGT “scars” associated with perchlorate reduction indicates that it is frequently propagated via horizontal gene transfer. This stands in stark contrast to reductive pathways such as denitrification, which are present in many different Proteobacteria and vary little in operon organization across broad phylogenetic groups.

The evolutionary trajectories of these two disparate metabolisms are likely shaped by the abundance of the respective electron acceptors in the environment. Denitrification is ubiquitous; nitrate is found in many types of aquatic and terrestrial environments at moderate concentrations and is continuously generated from inorganic nitrogen sources by nitrifying bacteria. Conversely, the are no known biological neogenesis mechanisms for perchlorate and it is rarely found in ecologically relevant quantities due to its slow abiotic generation in the atmosphere. The discrepancy between the abundance of these two compounds mimics the discrepancy in the frequency of the respective pathways in isolated bacteria.

This “rare perchlorate” hypothesis implicitly states that maintaining the PRI in the absence of perchlorate has a fitness cost. Although perchlorate is much more limited than nitrate in the environment, there are clearly situations where it is adaptive to have the ability to reduce perchlorate. Perchlorate accumulates in dry environments such as the Atacama Desert and the Antarctic Dry Valleys [45], but is also deposited across much wider geographic scales [1]. However, because its concentration in groundwater is tied to stochastic rainfall and irrigation events, many geographical areas with high washout may never reach a biologically relevant concentration of perchlorate, particularly prior to industrial contamination of perchlorate. These factors result in non-overlapping niches, or microenvironments where DPRB are successful but may be geographically isolated from other similar microenvironments, thus limiting their dispersion. In other words, we hypothesize that the frequency of environments where DPRB are successful is low enough or that selection against the PRI in non-perchlorate environments is negative enough, that the PRI is never fixed into a taxonomic group.

The stochastic pattern of natural perchlorate deposition and lack of continuous positive selection provides a plausible hypothesis for why an ancient metabolism would exist in such a low equilibrium abundance among bacterial genomes, yet continue to persist. However, release of anthropogenic perchlorate into the environment may be changing this equilibrium, providing a continuously selective environment for DPRB or increasing the abundance of PRIs globally. The minute number of SNPs among PRIs from the *Azospira* species relative to the rest of the respective genomes indicates a very recent acquisition of the PRI, especially relative to the divergence of the genomes as a whole. Further sequencing of genomes and PRIs of isolates and populations from contaminated sites, bioreactors, and pristine dry environments will be essential in gaining a more complete understanding of the dynamics of PRI evolution.

### Ancient evolution of perchlorate reduction

All extant sequences of PcrA and Cld are monophyletic and are linked together on the same “piece” of DNA in canonical DPRB. This suggests that all modern PRIs descended from a single progenitor PRI. Hypothetically, this progenitor PRI required three critical steps in its assembly. First and most important is the evolution of robust perchlorate reduction activity. Once an organism gains the ability to reduce perchlorate, the problem of reactive chlorine species presents itself. The chlorite dismutase enzymatic function likely predates the linkage of *cld* and *pcrA*, as there are Cld sequences basal to the Cld sequences found in DPRB. However, Cld sequences from DPRB are unique in the sense that they all contain a signal sequence indicating export to the periplasm. We hypothesize that this was another crucial step in the evolution of perchlorate reduction, which allowed chlorite to be dismutated near the site of its production in the periplasm. Once an organism acquired both of these abilities, it became the first perchlorate reducer. However, until these genes became genetically linked, this trait was likely inherited only vertically. The first PRI arose when *cld* and *pcrA* were incorporated on the same piece of DNA that could be transferred between unrelated organisms.

We propose that successful events of PRI horizontal gene transfer that lead to propagation within a new taxonomic group are rare. We also propose that less complex PRIs have a much better chance of being incorporated in taxonomically novel backgrounds; the acquisition of lineage-specific accessory genes comes later, once the PRI is being frequently transferred among phylogenetically similar organisms. An example of the early stages of this progress may be the PRIs in *Sedimenticola* spp. and *Arcobacter* sp. CAB which both contain transposase ‘scars’ within the PRI core (Fig. 4).

### Recent evolution of perchlorate reduction

Although extant lineages of perchlorate reduction derive from infrequent HGT events, our genomic data indicates that within these lineages recombination and transfer of the PRI is rampant. The most striking instance of this is the near-identity among the PRIs of the *Azospira* spp.

The fact that some of the accessory genes are pseudogenes also demonstrates the ongoing evolution of the PRI core (genes with dashed outlines, Fig. 4). Inactivation of genes by altering the start codon or introducing a nonsense mutation is an early step in this process, eventually followed by gene loss [46]. This likely occurs because genes in the PRI served an adaptive purpose in the donor organism, but are either neutral or deleterious in their current host [47].

## Conclusions

Perchlorate reduction is a unique metabolism that combines elements of aerobic and anaerobic respiration. The genomic basis for perchlorate reduction, the PRI, is a unique genomic island as well, encoding complex systems for regulation and oxidative stress response. We have shown in this study that the PRI is primarily associated with horizontal gene transfer on varying degrees of time scales. Understanding the dynamics of horizontal transfer of the PRI, as well as how it becomes integrated into host physiology, will be essential for understanding the ecology of this metabolism and where in the environment these genes exist. Towards this goal, we have integrated sequencing of taxonomically diverse isolates and genetic studies of a model system in order to understand the function of the genes in the PRI. We believe that perchlorate reduction provides an interesting model system for those looking at the intersection of microbial physiology and ecology, and how genomic organization and horizontally transferred genes shape microbial lifestyles and evolution.

## Additional files

Additional file 1: Table S2. List of locus tags for all of the genes mentioned explicitly or present in figures in this manuscript. (XLSX 53 kb) Additional file 2: Table S1. Information on all 13 genomes used in this manuscript. (XLSX 42 kb)

## Abbreviations

BLAST: Basic Local Alignment Search Tool
Cld: Chlorite dismutase
Contig: Contiguous region
Crp: Cyclic AMP receptor protein
CymA: Tetraheme cytochrome *c*
DMSO: Dimethyl sulfoxide
DPRB: Dissimilatory perchlorate-reducing bacteria
Fe-S clusters: Iron sulfur clusters
HGT: Horizontal gene transfer
ICE: Integrative and conjugative element
IspB: Octaprenyl diphosphate synthase
LysR: Lysine biosynthesis transcription regulator
MLSA: Multi-locus sequence analysis
MoaA: Molybdopterin biosynthesis protein A
Moco: Molybdenum cofactor
Mrp: Methionine rich peptide
Nap: Periplasmic nitrate reductase
Nrf: Periplasmic nitrite reductase
NrsF: Anti-sigma factor
Pcr: Perchlorate reductase
PilR: Two component histidine kinase response regulator
PilS: Two component histidine kinase sensor
PRI: Perchlorate reduction genomic island
SigF: Sigma factor
SNP: Single nucleotide polymorphism
XerD: Site specific tyrosine recombinase
YedYZ: Methionine sulfoxide reductase
